# Low Oxygen Levels Induce Early Luteinization Associated Changes in Bovine Granulosa Cells

**DOI:** 10.3389/fphys.2018.01066

**Published:** 2018-08-07

**Authors:** Vijay S. Baddela, Arpna Sharma, Torsten Viergutz, Dirk Koczan, Jens Vanselow

**Affiliations:** ^1^Reproductive Biology, Leibniz Institute for Farm Animal Biology (FBN), Dummerstorf, Germany; ^2^Institute of Immunology, University of Rostock, Rostock, Germany

**Keywords:** granulosa cells, oxygen levels, preovulatory follicle, gene expression, luteinization

## Abstract

During follicle maturation, oxygen levels continuously decrease in the follicular fluid and reach lowest levels in the preovulatory follicle. The current study was designed to comprehensively understand effects of low oxygen levels on bovine granulosa cells (GC) using our established estrogen active GC culture model. As evident from flow cytometry analysis the viability of GC was not found to be affected at severely low oxygen condition (1% O_2_) compared to normal (atmospheric) oxygen condition (21% O_2_). Estimations of hormone concentrations using competitive radioimmunoassay revealed that the production of estradiol and progesterone was significantly reduced at low oxygen condition. To understand the genome-wide changes of gene expression, mRNA microarray analysis was performed using Affymetrix’s Bovine Gene 1.0 ST Arrays. This resulted in the identification of 1104 differentially regulated genes of which 505 were up- and 599 down-regulated under low oxygen conditions. Pathway analysis using Ingenuity pathway analyzer (IPA) identified 36 significantly affected (*p* < 0.05) canonical pathways. Importantly, pathways like “Estrogen-mediated S-phase Entry” and “Cyclins and Cell Cycle Regulation” were found to be greatly down-regulated at low oxygen levels. This was experimentally validated using flow cytometry based cell cycle analysis. Up-regulation of critical genes associated with angiogenesis, inflammation, and glucose metabolism, and down-regulation of FSH signaling, steroidogenesis and cell proliferation indicated that low oxygen levels induced early luteinization associated changes in granulosa cells. Identification of unmethylated CpG sites in the *CYP19A1* promoter region suggests that granulosa cells were not completely transformed into luteal cells under the present low oxygen *in vitro* condition. In addition, the comparison with earlier published *in vivo* microarray data indicated that 1107 genes showed a similar expression pattern in granulosa cells at low oxygen levels (*in vitro*) as found in preovulatory follicles after the LH surge (*in vivo*). Overall, our findings demonstrate for the first time that low oxygen levels in preovulatory follicles may play an important role in supporting early events of luteinization in granulosa cells.

## Introduction

Ovaries are the female’s primary reproductive organs, which contain a large pool of primordial follicles. Under the influence of different endocrine factors, primordial follicles start maturation by forming an antrum that is filled with follicular fluid. The wall of antral ovarian follicles includes a peripheral vascularized theca cell layer which is separated from the inner avascular granulosa cell layer by a basement membrane (Siu and Cheng, 2013; Feng et al., 2017). Therefore, oxygen released from the capillaries will first reach the thecal cells. Lower amounts of oxygen will then diffuse through the basement membrane to reach the multiple layers of mural followed by antral GC and then to the cumulus –oocyte complex (COC). This clearly suggests that antral GC along with the COC are exposed to relatively low oxygen levels in large antral follicles. According to mathematic modeling, the dissolved concentrations of diffused oxygen in human ovarian follicular fluid are predicted between 11 and 51 mmHg, which corresponds to 1.5–6.7% of oxygen (Redding et al., 2008). Several other studies agree with these estimates and reported that the dissolved oxygen levels in follicular fluid is between 1 and 5% (Van Blerkom et al., 1997; Huey et al., 1999), which is far less than the atmospheric oxygen concentration (21%) that is generally used for culturing of follicular granulosa cells.

Granulosa cells are the major steroidogenic cells of ovarian follicles. During follicule maturation, follicle stimulating hormone (FSH) arouses steroid hormone production from the granulosa layer by inducing FSH receptor signaling (Rouillier et al., 1996). Once the follicle becomes dominant, an intense luteinizing hormone (LH) pulse from the pituitary gland increases the circulatory and follicular LH concentration, thus inducing early processes of luteinization and finally culminating in ovulation (Duffy and Stouffer, 2003). Dominant follicles will further increase in size during the post LH surge period, growing to a maximum diameter of 15–22 mm close to ovulation (Sartori et al., 2001; Christenson et al., 2013). As the diameter of ovarian follicles greatly increases during follicle maturation, the diffusion distance for gasses inside the follicle also increases. This will eventually lead to a continuous decrease of the oxygen concentration in the follicular fluid (Fischer et al., 1992). It has been already observed that falling pO_2_ is accompanied with a decrease of pH and an increase of pCO_2_ in the follicular fluid, indicating an active anaerobic respiration by follicular cells. These findings were further strengthened by the identification of increased glucose consumption and lactate accumulation in post hCG murine follicles (Harris et al., 2007). Recently, it was shown that even under normoxic conditions lactate can act as an effective signaling molecule that induces granulosa cell differentiation (Baufeld and Vanselow, 2018).

It is well known that cells of the granulosa and theca layers undergo early luteinization in preovulatory follicles immediately after the LH surge and display features that are very different from those found in dominant follicles before LH, and eventually become fully luteinized cells of the corpus luteum (Tesařík and Dvořak, 1982; Christenson et al., 2013). The most prominent changes associated with early luteinization of GC include a cease of GC proliferation, down-regulation of FSH signaling and steroidogenesis, and up-regulation of HIF1a signaling and angiogenesis (Christenson et al., 2013; Wissing et al., 2014). As the luteinization of GC commences within preovulatory follicles, we hypothesize that the prevailing low oxygen levels in preovulatory follicles may play a role in early luteinization of granulosa cells. Accordingly, the current study was carried out in our established estrogen active granulosa cell culture model to understand effects of low oxygen levels on the genome wide gene expression changes and steroid production in bovine granulosa cells.

## Materials and Methods

### Culturing of Granulosa Cells

Ovaries were collected at a commercial abattoir and granulosa cells were aspirated from small to medium follicles (≤6 mm) with a syringe and 18G needles. The number of viable cells was determined using the trypan blue exclusion method and the cells were eventually cryopreserved as described in a previous manuscript (Baufeld and Vanselow, 2013). All the chemicals for cell culture were purchased from Biochrom (Berlin, Germany) unless stated otherwise. Alpha (α)-MEM was enriched with supplements to make a working medium containing 0.1% BSA, 20 mM HEPES, 0.084% sodium bicarbonate, 2 mM Glutamin, 5 μg/ml transferrin, 4 ng/ml sodium selenite, 1 mM non-essential amino acids, 10 ng/ml insulin, 100 IU penicillin, and 0.1 mg/ml streptomycin. In addition, 20 ng/ml FSH (Sigma-Aldrich, Steinheim, Germany), 50 ng/ml IGF-I (Sigma-Aldrich, Steinheim, Germany) and 2 μM androstenedione (Sigma-Aldrich, Steinheim, Germany) were added to the α-MEM just before plating the cells. For experiments, the cryopreserved cells were rapidly thawed in a water bath, washed with α-MEM and plated at a density of ∼1.4 × 10^5^ viable cells per well in 24 well culture plate, which were pre-coated with collagen. The culture plates were kept in a CO_2_ incubator at 21% O_2_ and 5% CO_2_ during the next 6 days, with media exchange every 48 h. On the 6th day, cells were incubated at low oxygen (1% O_2_) and normal oxygen conditions (21% O_2_), separately, for the next 48 h to analyze the low oxygen induced effects.

### Cell Viability and Apoptosis Analysis

On the 8th day of culture, spent culture media were collected in a 1.5 ml collection tube and centrifuged to pellet the floating dead cells. The attached cells in the culture plate were washed twice with PBS and detached by adding 250 μl of tryplE solution (Thermo Fischer, United States) to each well of the 24 well plates. The detached cells were added to the above pelleted cells to ensure the inclusion of floating and attached cells into the analysis. The cells were pelleted and washed using 1 ml of α-MEM and subjected to viability and apoptosis analysis using the Annexin-V FITC kit (Miltenyi Biotec, Germany). Briefly, the cell pellet was re-suspended in 100 μl of 1× binding buffer followed by adding 10 μl of Annexin V reagent. After gentle mixing, the tubes were incubated in the dark for 15 min. Cells were washed and re-suspended in 500 μl of 1× binding buffer. Then 5 μl of propidium iodide (PI) was added to the cells and mixed gently just before the flow cytometry analysis. The fluorescence signal was quantified from single cells (10,000 counts) using a flow cytometer (Gallios, Beckman-Coulter, Germany) and the data were analyzed using the Kaluza-software (Beckman-Coulter, Germany).

### RNA Isolation, cDNA Preparation, qPCR Analysis

Total RNA was isolated using the Nucleo Spin RNA II Kit (Macherey-Nagel, Düren, Germany) by following the manufacturer’s instructions. RNA was quantified using a NanoDrop 1000 spectrophotometer (Thermo Scientific, Bonn, Germany) and cDNA was prepared using the SensiFast cDNA synthesis kit (Bioline, Luckenwalde, Germany). The gene expression analysis for the selected genes was performed using the SensiFast SYBR No-ROX (Bioline) reagent and gene specific primers (**Supplementary Data Sheet S1**) in a Light Cycler 96 instrument (Roche, Mannheim, Germany). For qPCR analysis, two different volumes (2 and 4 μl) of cDNA were amplified in 12 μl total reaction volume. The qPCR cycling conditions used are shown in **Supplementary Data Sheet S2**. Amplicons from all analyzed genes, were cloned in PGEM-T vectors (Promega Biosciences, United States) and sequenced to verify the product. External standard curves were generated during each run from plasmids at five different serially diluted concentrations (5 × 10^-12^ to 5 × 10^-16^ g plasmid). The Abundance of transcripts was normalized using TBP as a validated house-keeping gene under low oxygen conditions (Baddela et al., 2014).

### Microarray Analysis

To identify changes of the global gene expression profiles induced by low oxygen levels, total RNA was isolated from GC cultured under normal (NOL 1–4) and low oxygen levels (LOL 1–4) and analyzed using GeneChip^TM^ Bovine Gene 1.0 ST Array (Affymetrix^®^, Inc., Santa Clara, CA, United States). RNA integrity was measured in a bio analyzer instrument, which revealed RIN values from 9.7 to 10 in all samples (**Supplementary PDF File S1**). The subsequent amplification and labeling of RNA samples was performed using GeneChip 3′ amplification and one-cycle target labeling reagents. Overnight hybridization of RNA samples and probes was carried out in a hybridization oven followed by acquisition of the gene expression signals using an Affymetrix Gene Chip Scanner 3000. Normalization and background reduction of gene expression was performed using the robust multichip average method. The acquired data were subsequently analyzed using the TAC 4.0 software (Transcriptome Analysis console 4.0, Affymetrix). Differentially expressed (DE) genes were recognized using the cut off parameters, fold difference >| 2|, ANOVA *p* < 0.05, and FDR (*q*) < 0.05.

### Estimation of Estradiol and Progesterone Concentration

Estradiol (E2) and progesterone (P4) concentrations were estimated in the spent media using a sensitive single antibody ^3^H-radioimmunoassay performed in a competitive mode. The antibodies were raised in rabbit and purified using affinity chromatography. The E2 tracer, (2,4,6,7-3H estradiol-17β), was purchased from GE Healthcare (Freiburg, Germany) and P4 tracer, [1,2,6,7-3H(N) progesterone], was purchased from PerkinElmer (Boston, United States). Assay standards were prepared in RIA buffer after dissolving the tracers in 100% ethanol. The levels of radioactivity were measured in a liquid scintillation beta-counter containing an integrated RIA-calculation program (TriCarb 2900 TR; PerkinElmer, Germany).

### Determination of Cell Proliferation

Cell proliferation was analyzed by identifying the number of cells in different phases of the cell cycle using flow cytometer analysis. GC were cultured for 8 days as described above. On the 8th day, spent culture media were collected in a 1.5 ml collection tube and centrifuged to pellet floating cells. The cells were washed twice using 1× PBS and then detached from the plate by adding 250 μl of tryplE reagent (Thermo Fisher, United States) to each well. The detached cells were added to the floating cells of the corresponding wells. Cells were pelleted, washed and dissolved in 300 μl of 1× PBS. The cell suspension was subsequently added dropwise into 70% ice cold ethanol and stored at -20°C for 2 h. Then, cells were centrifuged (300 × *g*, 10 min, 4°C), re-suspended in 1 ml RNase solution (1 mg/ml) and incubated at 37°C for 30 min. The PI reagent (final concentration 50 μg/ml) was added to the cells and incubated for 30 min at 37°C in the dark. The fluorescence was quantified from single cells (10,000 counts) using a flow cytometer (EPICS-XL, Beckman-Coulter, Krefeld, Germany). The data were subsequently analyzed using the Multicycle software (Phoenix, United States).

### Methylation Analysis of CpG Sites in *CYP19A1* Promoter 2.0

Methylation of *CYP19A1* at three CpG dinucleotide positions -35, +18, and +30, relative to the GC-specific start site of transcription, in the proximal promoter 2.0 region were analyzed using the bisulfite direct sequencing method. Genomic DNA was isolated from GC cultured under normal oxygen (*n* = 5) and low oxygen (*n* = 5) conditions and modified using the EZ DNA Methylation-Gold kit (Zymo, Freiburg, Germany). PCR was performed using HotStarTaq Plus reagents (Qiagen, Hilden, Germany) and gene specific primers (**Supplementary Data Sheet S1**) at following cycling conditions: pre-incubation at 95°C for 5 min; 40 cycles of denaturation at 95°C for 75 s, annealing at 53°C for 75 s, and extension at 72°C for 35 s. PCR products were analyzed by agarose gel electrophoresis (3%, ethidium bromide stained) and purified using the High Pure PCR Purification Kit (Roche). Sequencing of PCR products was performed at the institutional core facility. The sequence files were evaluated using a Web based software QUMA (QUantification tool for Methylation Analysis), available at http://quma.cdb.riken.jp/top/index.html, to quantify the percent of methylated vs. un-methylated cytosine nucleotides at individual CpG dinucleotides.

### Bioinformatics and Statistical Analysis

All bioinformatic analyses were carried out for the human homologs of DE genes. The enriched gene ontology terms were recognized using WebGestalt, a WEB based gene set analysis tool kit. The canonical pathways and upstream regulators were identified using the Ingenuity pathway analysis tool (IPA, Qiagen, Hilden). Further, hub genes were recognized by constructing a protein-protein interaction network using NetworkAnalyst tool available at www.Networkanalyst.ca. Microarray data analysis was performed using integrated statistical measures available in TAC 4.0 software. Analysis of Variance (ANOVA) was used to calculate the *p*-values, which were further corrected by False Discovery Rate (Benjamini–Hochberg method) measures. Significance levels were set at fold change > |2|, ANOVA *p* < 0.05, and FDR (*q*) < 0.05 for identifying DE genes. The qPCR gene expression, RIA and flow cytometry values were analyzed by using *t*-test in GraphPad prism 5.0 software. Significant changes were acknowledged if *p* < 0.05.

## Results

### Effect of Low Oxygen Levels on the Viability and Steroidogenesis of Granulosa Cells

**FIGURE 1:**
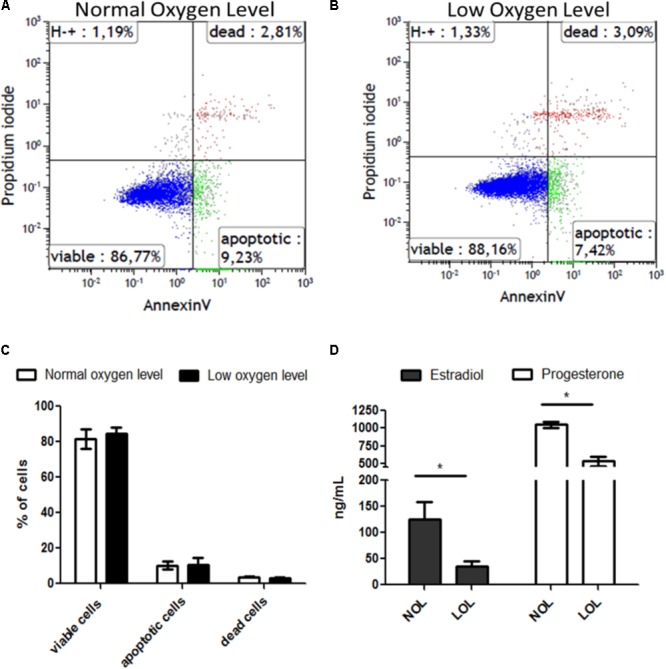
Effect of low oxygen levels on the viability and steroidogenic capacity of granulosa cells. **(A,B)** Visualize representative histograms of cells treated with normal and low oxygen levels, respectively, in flow cytometry analysis. **(C)** Means ± SEM of three independent experiments are represented. **(D)** Estradiol (black bars) and progesterone (white bars) concentrations are shown at low (LOL) and normal oxygen levels (NOL). Results are means ± SEM of three independent experiments. Significant changes were acknowledged with asterisks if *p* < 0.05 in *t*-testing.

After subjecting GC to low and normal oxygen levels (**Figure 1** and **Supplementary Figure S1**), the percentage of live, apoptotic and dead cells was determined using flow cytometric analysis by adding propidium iodide (PI) and annexin-V reagents to the detached cells. This revealed that GC did not show significant variation in healthy viable (PI-, Annexin-), apoptotic (PI-, Annexin+) and dead (PI+, Annexin+) cell counts at low oxygen levels compared to cells grown at normal oxygen levels (**Figure 1C**). However, unlike the viability status of the cells, levels of estradiol and progesterone were significantly reduced at low oxygen levels (**Figure 1D**).

### Microarray Data

**FIGURE 2:**
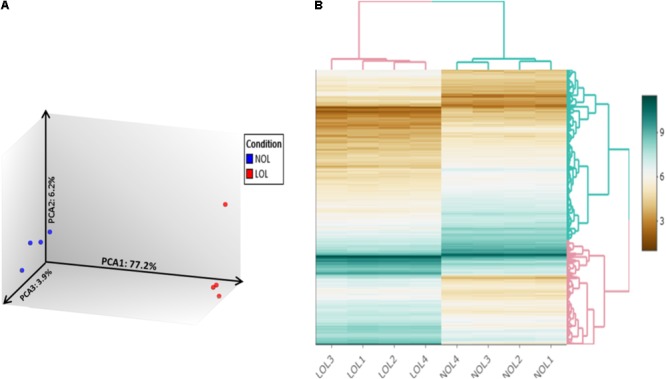
Principle component analysis and clustered heat mapping. Unsupervised principal component analysis visualizes differences in the transcriptomes of bovine GC cultured at normal and low oxygen conditions **(A)**. Blue and red color dots denote individual samples from normal and low oxygen conditions, respectively. Each axis indicates the fraction of percentage of variation out of total mapped variation among samples. **(B)** Hierarchically clustered heatmap of differentially expressed genes between samples treated with normal (NOL1–NOL4) and low oxygen levels (LOL1–LOL4).

Raw microarray data files were analyzed using the TAC 4.0 software. Evaluation of data from 3′ and 5′ hybridization controls (**Supplementary Figure S2**) and normalized signal box plots (**Supplementary Figure S3**) indicated that all array files were normal and passed the quality checkup (QC). Subsequent principal component analysis (PCA) of the microarray data sets (**Figure 2A**) showed that the samples from low and normal oxygen treatments were located most distant from each other with a variation of 77.2% on principal component axis 1 (PCA1), thus indicating the remarkable differences of the gene expression profiles between the two treatments. Further, a mere variation of 6.3 and 3.9% was observed on the PCA2 and PCA3 axes, respectively, which is mainly due the variation between samples treated with low or normal oxygen concentrations.

A total of 20422 publicly annotated gene clusters (**Supplementary Data Sheet S3**) were identified in the microarray data. Among them, 1104 genes were recognized to be differentially expressed (FC > | 2| ; *p* < 0.05 and FDR < 0.05) between the GC cultured at low and normal oxygen levels (**Supplementary Data Sheet S4**). Specifically, 505 and 599 genes were up- and down-regulated, respectively, at low oxygen levels. The abundance of DE genes was visualized in the form of an interactive heat map (with zoom in and out features), which was constructed using shinyHeatmaply package in R studio (**Supplementary Html File S1**). The same heat map is shown in a static form in **Figure 2B**. The top twenty down- and up- regulated genes at low oxygen levels are listed in **Tables 1, 2**.

**Table 1:** Top twenty down-regulated genes at low oxic conditions.

Affymetrix ID	Gene symbol	Description	MES at NOL	MES at LOL	FC (NOL/LOL)
12837074	*TXNIP*	Thioredoxin interacting protein	190.01	17.14	11.06
12836232	*LRP8*	Low density lipoprotein receptor-related protein 8, apolipoprotein e receptor	369.64	45.25	8.18
12688063	*CYP19A1*	Cytochrome P450, family 19, subfamily A, polypeptide 1	413.00	54.56	7.56
12849517	*GPR85*	G protein-coupled receptor 85	25.45	3.55	7.15
12818766	*LOC104976005*	Uncharacterized LOC104976005	108.38	16.56	6.53
12786691	*LOC783613*	Dynein light chain 1, cytoplasmic	32	5.31	6.02
12802575	*LOC527388*	Histone H4	93.05	16.11	5.77
12704737	*RRM2*	Ribonucleotide reductase M2	59.71	10.33	5.75
12846849	*LOC786781; CTH*	Cystathionine gamma-lyase-like; cystathionine gamma-lyase	55.33	9.78	5.64
12774048	*RCC2; MIR2358*	Regulator of chromosome condensation 2; microRNA mir-2358	49.52	9.18	5.39
12901592	*CENPW*	Centromere protein W	9.31	1.85	5.01
12800906	*LOC104968446*	Histone H2A type 1	445.72	89.26	4.98
12697409	*ARHGAP11A*	Rho GTPase activating protein 11A	33.12	6.72	4.92
12904800	*GPR50*	G protein-coupled receptor 50	148.05	30.27	4.89
12882714	*CTH*	Cystathionine gamma-lyase	55.33	11.47	4.84
12902815	*RGN*	Regucalcin	92.41	19.15	4.82
12715265	*SLC2A10*	Solute carrier family 2 (facilitated glucose transporter), member 10	83.86	17.63	4.77
12869943	*CCNA2*	Cyclin A2	74.02	16	4.65
12735060	*SRM*	Spermidine synthase	160.89	35.75	4.5
12871633	*ELOVL6*	ELOVL fatty acid elongase 6	352.13	79.34	4.42

*MES, mean expression signal; FC, fold change.*

**Table 2:** Top twenty up-regulated genes at low oxygen conditions.

Affymetrix ID	Gene symbol	Description	MES at NOL	MES at LOL	FC (NOL/LOL)
12812382	*HBA; HBA1*	Hemoglobin, alpha 2; hemoglobin, alpha 1	11.39	2721.14	–239.21
12862249	*BHLHE41*	Basic helix-loop-helix family, member e41	53.07	1144.10	–21.58
12822964	*PPP1R3C*	Protein phosphatase 1, regulatory subunit 3C	13.54	272.47	–20.04
12846870	*CIART*	Circadian associated repressor of transcription	15.56	261.37	–16.81
12900682	*VNN1*	vanin 1	20.25	302.33	–14.97
12776671	*LIMS2*	LIM and senescent cell antigen-like domains 2	22.47	312.99	–13.9
12894529	*LOXL2*	Lysyl oxidase-like 2	33.82	436.54	–12.94
12733712	*BDNF*	Brain-derived neurotrophic factor	11.63	141.04	–12.15
12864766	*KRT18*	Keratin 18	43.11	522.75	–12.09
12721948	*NDRG1*	N-myc downstream regulated 1	153.27	1833.01	–11.96
12798624	*PARP3*	Poly (ADP-ribose) polymerase family, member 3	18.76	219.79	–11.67
12850003	*FGL2*	Fibrinogen-like 2	12.99	139.10	–10.71
12766049	*NLGN2*	Neuroligin 2	45.88	477.71	–10.39
12723335	*CALB1*	Calbindin 1, 28kDa	7.31	74.54	–10.14
12889942	*ZNF395*	Zinc finger protein 395	44.63	430.53	–9.65
12727781	*TAGLN*	Transgelin	46.20	418.76	–9.09
12837477	*ACKR3*	Atypical chemokine receptor 3	35.01	298.17	–8.52
12881035	*PRR7*	Proline rich 7 (synaptic)	19.97	166.57	–8.32
12741966	*CABP1*	Calcium binding protein 1	7.16	58.08	–8.11
12903629	*RAB33A*	RAB33A, member RAS oncogene family	16.44	133.43	–8.09

*MES, mean expression signal; FC, fold change.*

Particularly, *TXNIP* (thioredoxin interacting protein) showed strongest down-regulation (FC = 11.06; *q* = 4.12E-05), whereas *HBA*, encoding hemoglobin alpha chain, showed strongest up-regulation (FC = 239.21; *q* = 3.04E-11) under low oxygen conditions. Other important changes comprised down-regulation of granulosa cell marker genes, *FOXL2* (FC = 3.48; *q* = 1.08E-06), *FSHR* (FC = 3.17; *q* = 8.66E-08) and *CYP19A1* (FC = 7.56; *q* = 6.21E-09) and up-regulation of genes associated with angiogenesis, *VEGFA* (FC = 3.37; *q* = 3.46E-08), *VEGFB* (FC = -2.21; q = 1.91E-06), *VCAM1* (FC = -3.91; *q* = 2.93E-06), *EDNRA* (FC = -2.1; *q* = 0.0001), *ANGPT2* (FC = -5.63; *q* = 2.96E-07) and *ANGPTL4* (FC = -4.86; *q* = 5.90E-08), and inflammation related genes, *PTGES* (FC = -5.23; *q* = 6.12E-08), *VNN2* (FC = -4.14; *q* = 1.50E-07) and *VNN1* (FC = -14.97; *q* = 3.80E-09). Further on, multiple uncharacterized genes (*LOC104976005, LOC783613, LOC527388, LOC786781358*, and *LOC104968446* etc.) were found to be regulated by differential oxygen concentrations. However, their functions with respect to granulosa and luteal cell function is not yet known.

**FIGURE 3:**
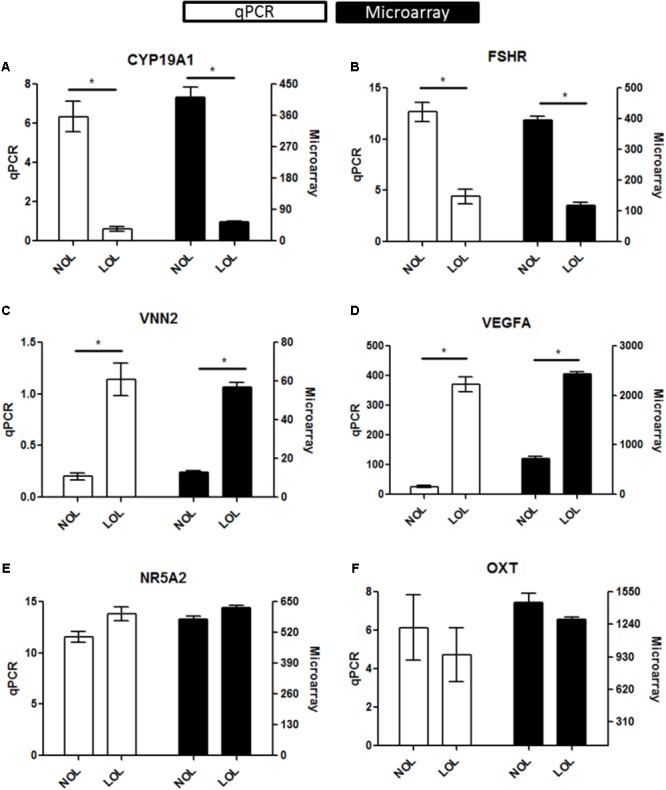
qPCR evaluation of microarray data: Diagrams **(A–F)** represent data of selected marker genes *CYP19A1, FSHR, VNN2, VEGFA, NR5A2*, and *OXT*. The left vertical axis indicates the qPCR gene expression values (Mean ± SEM), whereas the right vertical axis indicates the linear hybridization signal values from microarray data. On the X-axis, NOL indicates normal oxygen and LOL low oxygen level condition. Significant changes (^∗^) were acknowledged if *p* < 0.05 for qPCR values in *t*-testing. Significance was attributed to microarray signals if the Fold change > |2|, ANOVA *p* < 0.05, and FDR *q* < 0.05.

Microarray data were validated using RNA samples isolated from three independent experiments, different from those used for microarray analysis. A total of six genes were selected for re-assessing the expression values. These include, up-regulated (*VNN2* and *VEGFA*), down-regulated (*FSHR* and *CYP19A1*) and un-regulated genes (*NR5A2* and *OXT*). The normalized expression of all these genes was found to be similar in both qPCR and microarray estimations (**Figure 3**).

### Bioinformatics Interpretations

**FIGURE 4:**
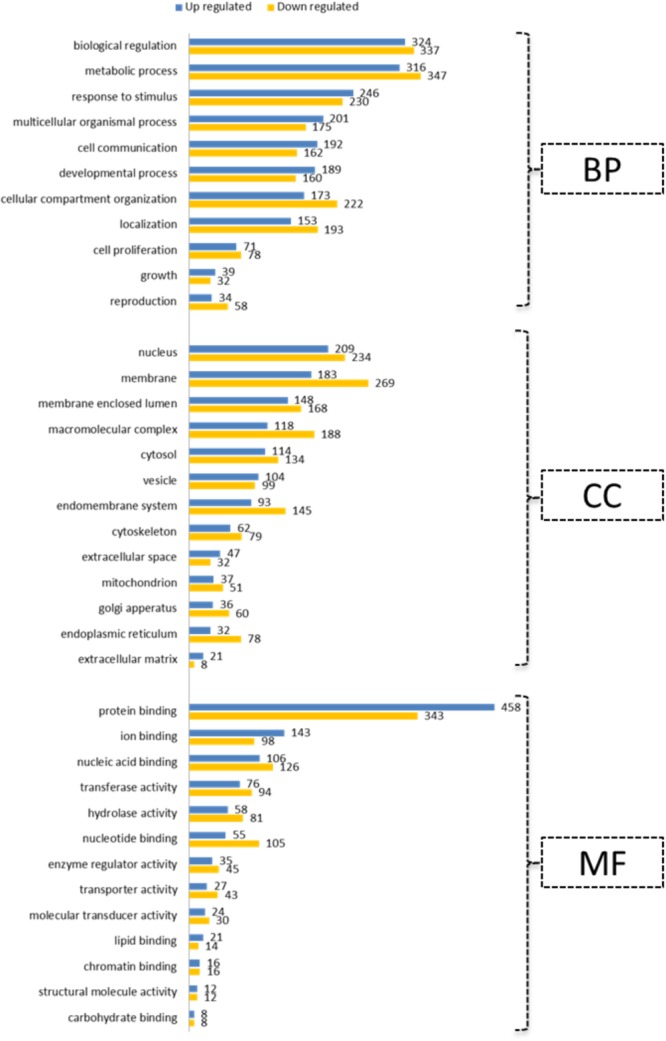
Gene ontology annotations. Up- and down-regulated genes under low oxygen conditions were subjected to GO slim annotations. The submitted genes were classified into categories of 11 biological processes (BP), of 13 cell components (CC) and of 13 molecular functions (MF). Vertical axes indicate prioritized functions and horizontal axes indicate the number of genes associated with each of the represented annotations.

All bioinformatic analyses were performed for the human homologs of DE genes. Initially, GO terms were generated separately for up- and down-regulated genes using the WebGestalt tool (**Figure 4**). This indicated the prioritized list of biological processes, cell components and molecular function categories for genes regulated by differential oxygen levels. For Example: 192 and 71 up-regulated genes are involved in “cell communication” and “cell proliferation,” respectively, at LOL compared to 162 and 78 genes (down-regulated at LOL) at NOL, respectively. Likewise, genes localized in the nucleus are prioritized at LOL whereas membrane proteins are prioritized at NOL. There were 21 genes identified to have extracellular matrix functions at LOL compared to eight genes at NOL. Similar observations can also be noticed with respect to different molecular functions at different oxygen levels. Further, to understand the detailed functional changes induced by differential oxygen concentrations, enriched canonical pathways were identified using IPA. Among 283 different canonical pathways identified by IPA (**Supplementary Data Sheet S5**), 36 were significantly enriched (*p* < 0.05) and among them 17 were showing either positive or negative *z* score values (**Table 3**), which indicate the propensity of a particular pathway under given treatments.

**Table 3:** List of canonical pathways enriched for differentially expressed genes in IPA.

Ingenuity canonical pathways	*P*-value	*z*-score	Molecules
Estrogen-mediated S-phase entry	8.71E-06	3	CCNA2/E2F4/CCNE2/CDKN1A/CDKN1B/ESR1/ CDK1/CDC25A/SKP2
Mitotic roles of polo-like kinase	0.000135	2.53	KIF23/PLK4/CDC20/TGFB1/PTTG1/PRC1/CDC16/ CDK1/KIF11/CCNB1/CDC25A/SMC1A
Cyclins and cell cycle regulation	0.000457	3.317	CCNA2/E2F4/CCNE2/CCND2/TGFB1/HDAC11/ CDKN1A/TGFB2/CDKN1B/CDK1/CCNB1/SKP2/CDC25A
Aryl hydrocarbon receptor signaling	0.000537	3.051	GSTA3/CCNE2/NQO1/APAF1/CYP1B1/CHEK1/ CCNA2/JUN/CCND2/TGFB1/RARA/NEDD8/ CDKN1A/TGFB2/HSPB7/DHFR/CDKN1B/ESR1
Cell cycle: G2/M DNA damage checkpoint regulation	0.001778	–2.333	TOP2B/GADD45A/CDKN1A/TOP2A/BORA/ CDK1/CHEK1/CCNB1/SKP2
Retinoic acid Mediated apoptosis signaling	0.003162	0.378	TIPARP/RARA/ZC3HAV1/APAF1/CYCS/ PARP3/CRABP2/TNFRSF10A
Cell cycle: G1/S checkpoint regulation	0.004677	–1.89	E2F4/CCNE2/CCND2/TGFB1/HDAC11/ CDKN1A/TGFB2/CDKN1B/CDC25A/SKP2
p53 signaling	0.004898	–1.265	PIK3R3/PCNA/CCND2/JUN/GADD45A/THBS1/PIK3CG/ PIK3R1/CDKN1A/PIAS1/APAF1/TNFRSF10A/BIRC5/CHEK1
AMPK signaling	0.006761	–0.728	PFKFB3/RAB27A/CPT1A/SLC2A1/RAB3A/PIK3R1/CPT1B/ PFKL/PFKP/EIF4EBP1/PIK3R3/CCNA2/ GYS1/PFKFB4/FASN/PIK3CG/CDKN1A/PRKAA2/AK4/ ACACA/HMGCR/PHF10
Interferon signaling	0.010233	2.449	SOCS1/OAS1/PTPN2/MX1/PIAS1/TAP1
ATM signaling	0.012882	–0.302	JUN/SMC2/GADD45A/FANCD2/H2AFX/CDKN1A/ZEB1/CDK1/ CHEK1/CCNB1/CDC25A/SMC1A
Type I diabetes mellitus signaling	0.0302	1	TRAF6/SOCS1/SOCS3/PIAS1/HLA-B/APAF1/CYCS/ SOCS4/HLA-DQB1/IL1R1/MAP3K5
SAPK/JNK signaling	0.034674	0.302	MAP4K2/PIK3R3/MAP3K9/JUN/GADD45A/PIK3CG/ MAP3K13/PIK3R1/DUSP10/MAP3K5/MAP3K2
CD27 signaling in lymphocytes	0.040738	–1.633	MAP3K9/JUN/MAP3K13/APAF1/ CYCS/MAP3K5/MAP3K2
Death receptor signaling	0.041687	0.632	ACTA2/TIPARP/ZC3HAV1/APAF1/CYCS/ HSPB7/ACTG2/PARP3/MAP3K5/TNFRSF10A
Role of CHK proteins in cell cycle checkpoint control	0.044668	–0.447	PCNA/E2F4/CDKN1A/CDK1/ CHEK1/RAD1/CDC25A
NRF2-mediated oxidative stress response	0.046774	–0.816	GSTA3/PIK3R1/NQO1/DNAJC3/DNAJC10/JUNB/MAP3K5/MAFF/ PIK3R3/HMOX1/JUN/ACTA2/DNAJB11/PIK3CG/ACTG2/ FKBP5/HACD3

Importantly, pathways associated with cellular proliferation including Estrogen-mediated S-phase Entry (*p* = 8.70964E-06), Cyclins and Cell Cycle regulation (*p* = 0.000457088), Cell Cycle: G2/M DNA Damage Checkpoint Regulation (*p* = 0.001778279), and Cell Cycle: G1/S Checkpoint Regulation (*p* = 0.004677351) were strongly affected by the oxygen concentration in granulosa cells.

**FIGURE 5:**
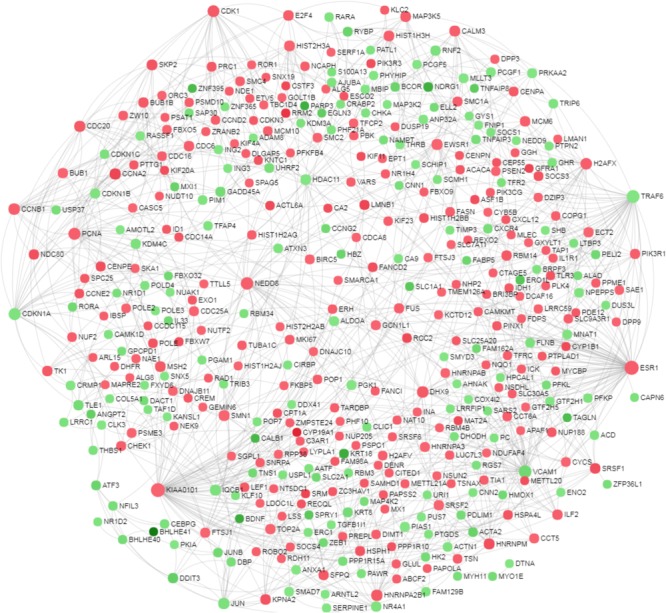
Network based meta-analysis of differentially expressed genes. A zero order protein-protein interaction network was identified among the differentially expressed genes of the microarray data to recognize the critical hub genes. The nods with green and red color indicate down- and up-regulated transcripts under low oxygen conditions.

Further, a zero order PPI network was identified among the DE genes to recognize the critical hub genes affected by the oxygen concentration. The resultant network contained 414 nodes with 874 connecting edges (**Figure 5** and **Supplementary Data Sheet S6**). The hub genes were ranked based on their interacting degree and betweenness in the PPI network. Importantly, *ESR1* was identified to be the most highly ranked hub gene with a degree = 70 and betweenness = 20183. It is followed by *KIAA0101* with a degree = 65 and betweenness = 16119. The top 20 ranked hub genes in the PPI network along with their degree and betweenness of interaction were mentioned in the **Table 4**.

**Table 4:** List of hub genes.

Gene symbol	Description	Degree	Betweenness	FC (NOL/LOL)
*ESR1*	Estrogen receptor 1	70	20183.19	2.19
*KIAA0101*	KIAA0101 ortholog	65	16118.99	2
*TRAF6*	TNF receptor-associated factor 6, E3 ubiquitin protein ligase	42	14937.06	–2.15
*VCAM1*	Vascular cell adhesion molecule 1	38	7215.39	–3.91
*NEDD8*	Neural precursor cell expressed, developmentally down-regulated 8	35	7411.9	2.25
*CDKN1A*	Cyclin-dependent kinase inhibitor 1A (p21, Cip1)	28	8569.35	–2.38
*PCNA*	Proliferating cell nuclear antigen	28	6574.45	2.35
*CDK1*	Cyclin-dependent kinase 1	25	5767.09	2.72
*H2AFX*	H2A histone family, member X	22	3622.03	2.51
*DHX9*	DEAH (Asp-Glu-Ala-His) box helicase 9	19	3123.61	2.72
*CCNB1*	cyclin B1	19	3076.66	2.3
*HNRNPA2B1*	Heterogeneous nuclear ribonucleoprotein A2/B1	18	3934.21	2.62
*JUN*	Jun proto-oncogene	17	5189.28	–2.88
*CCNA2*	Cyclin A2	16	2692.33	4.65
*EWSR1*	EWS RNA-binding protein 1	16	2686.22	2
*CDC20*	Cell division cycle 20	16	1854.56	2.74
*CALM3*	Calmodulin 3 (phosphorylase kinase, delta)	14	4101.26	2.28
*IQCB1*	IQ motif containing B1	14	1982.65	–2.88
*SMN2*	Survival of motor neuron 2, centromeric	14	1913.5	2.01
*HNRNPM*	Heterogeneous nuclear ribonucleoprotein M	14	1362.04	3.46

### Low Oxygen Levels Affect Cellular Proliferation

**FIGURE 6:**
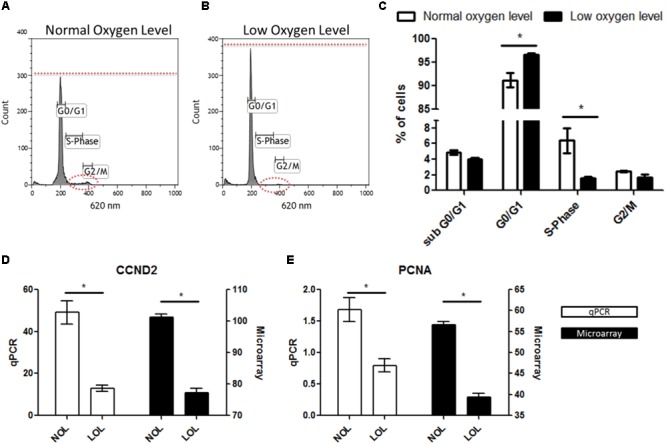
Effects of low oxygen levels on granulosa cell proliferation. **(A,B)** Represent flow cytometry histograms of PI stained granulosa cells after normal and low oxygen level treatments, respectively. Differences in the relative fluorescence at G0/G1, S and G2/M phases were marked with dotted lines. **(C)** Mean ± SEM values of three independent experiments showing numbers of cells in different phases of the cell cycle. **(D,E)** Transcript abundance of *CCND2* and *PCNA* in granulosa cell at normal and low oxygen conditions. Significant differences (asterisks) were acknowledged when *p* < 0.05 in *t*-testing.

Ingenuity pathway analyzer analysis revealed that pathways related to the cell cycle were found to be majorly affected under low oxygen concentration. To validate this finding, GC were analyzed in a flow cytometer to determine the percent of cells in different stages of the cell cycle. Confirming the IPA analysis, flow cytometry analysis showed that GC were significantly arrested in G0/G1 phase with significantly less numbers of cells undergoing cellular proliferation at LOL (**Figures 6A,B**). Specifically, 96.68 ± SEM 0.3% of GC underwent the cell cycle arrest in the G0/G1 phase, which further ceases the cellular replication as only 1.6 ± SEM 0.1% of cells were found to be in the S phase of the cell cycle at LOL (**Figure 6C**). Whereas 91.13 ± SEM 1.5% and 6.4 ± SEM 1.6% of cells were found to be in the G0/G1 phase and S-Phase of the cell cycle, respectively, at normal oxygen conditions. No significant differences were observed in the number of cells at sub G0/G1 and G2/M phases at low and normal oxygen treatments. Further, qPCR analysis of proliferation markers, CCND2 (**Figure 6D**) and PCNA (**Figure 6E**), showed that low oxygen levels significantly down-regulated the expression of these marker transcripts, which in turn supports the data of flow cytometer and IPA analysis.

### Methylation Analysis of the *CYP19A1* Proximal Promoter P 2.0

**FIGURE 7:**
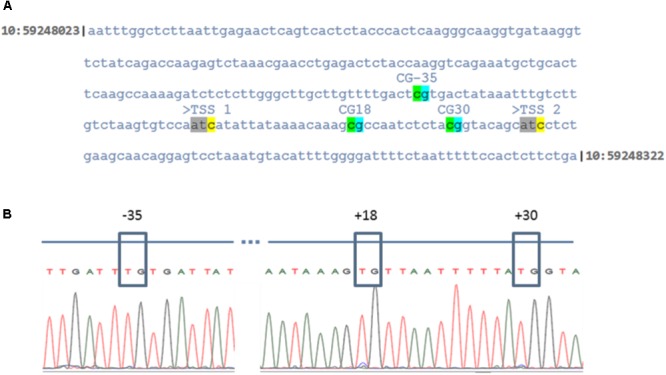
DNA methylation levels of the proximal *CYP19A1* promoter region in granulosa cells at low and normoxic conditions. **(A)** Indicates the sequence of genomic DNA corresponding to the *CYP19A1* proximal promoter region on chromosome 10 between the genomic region, 59248023 to 59248322. The three CpG sites with respect to the main ovarian transcription start site (TSS 2) are highlighted. **(B)** Electropherogram of the bisulfite treated and sequenced *CYP19A1* promoter region. The CpG sites were boxed to show that cytosine residues were unmethylated in all samples irrespective of differential oxygen treatment.

The present microarray and qPCR data revealed that *CYP19A1* expression is down-regulated in GC cultured at low oxygen conditions. It has been shown that the *CYP19A1* gene is methylated at three CpG sites that are present in the proximal promoter region at positions -35, +18 and +30 in granulosa derived luteal cells *in vivo* (Vanselow et al., 2005, 2010). Therefore, these individual CpG nucleotides were analyzed as a marker for complete luteinization in cultured GC at low and normal oxygen levels. Sequencing of bisulfite modified DNA revealed that these CpG sites are completely unmethylated (100%) in all 10 candidate DNA samples, isolated from independently cultured granulosa cells. The corresponding sequence of the *CYP19A1* proximal promoter and chromatogram of sequenced but modified DNA are shown in **Figure 7**.

## Discussion

Ovarian follicles are highly dynamic structures, which undergo sequential maturation, ovulation and luteinization processes during a successful reproductive cycle. Due to the lack of direct blood supply, pO_2_ in the follicular fluid is found to decrease during follicular maturation, reaching lowest levels in preovulatory follicles (Fischer et al., 1992). Increased accumulation of lactate and of the hypoxia inducible factor 1a (HIF1a) in the follicular fluid additionally indicate the existence of low oxygen conditions in ovarian follicles (Harris et al., 2007; Duncan et al., 2008; Kim et al., 2009). In the present study, the observed absence of significant changes in the viability and apoptotic status of GC at LOL compared to NOL intuitively indicated that GC might be naturally adapted to survive under low oxygen levels.

However, contrasting observations were reported in other cell types in which apoptosis was induced by low oxygen levels (Zheng et al., 2012). Further, by analyzing the mRNA transcriptome, we could show that low oxygen levels alter the gene expression profile of granulosa cells in a highly specific manner.

### Low Oxygen Levels Induce Early Luteinization Associated Changes of the Transcriptome in Granulosa Cells

A clear separation of samples treated with low and normal oxygen levels on PCA1 of principal component analysis visibly indicated that the oxygen concentration could remarkably affect the global gene expression profile in granulosa cells. The subsequent data analysis resulted in the identification of 1104 differentially expressed genes under low oxygen concentrations. Importantly, genes associated with FSH signaling, which include *FSHR, CYP19A1*, and *LHCGR* were significantly down-regulated at low oxygen levels. FSH signaling is a characteristic phenotypic feature of granulosa cells (Mihm and Evans, 2008). It induces the expression of *CYP19A1*, which in turn is involved in the production of estradiol from the theca layer derived androstenedione (Parakh et al., 2006). The remarkable down-regulation of *CYP19A1* expression at low oxygen level was further reflected by reduced estradiol production. Our group has earlier reported that low-level *CYP19A1* expression in granulosa lutein cells of mature CL coincides with methylation of the main ovarian *CYP19A1* promoter P 2.0 (Vanselow et al., 2006, 2010). Therefore we analyzed the methylation levels of individual CpG dinucleotides within the *CYP19A1* promoter P 2.0. The present data, however, indicated that hypoxic culture conditions could not induce increased DNA-methylation levels in GC-specific *CYP19A1* promotor 2.0. This could be possibly due to the fact that the cultured GC were not completely transformed into luteal cells under low oxygen conditions.

It is well known that *FOXL2* (Forkhead box protein L2) plays an important role in upholding GC identity (Georges et al., 2014). Genomic deletion of *FOXL2* leads to the development of seminiferous tubules like structure in female mice (Uhlenhaut et al., 2009). *FOXL2* levels were found to be down-regulated in preovulatory granulosa cells and in the corpus luteum (Pisarska et al., 2004). During our study we found down-regulation of *FOXL2* under hypoxic condition in GC thus suggesting that the cells are driven toward luteinization by low oxygen levels.

Contrary to FSH signaling, GC cultured at LOL showed a significant up-regulation of multiple genes (*VEGFA, TGFB2, VCAM1, VEGFB, ENDRA, ANGPT2*, and *ANGPTL4*) involved in angiogenesis and endothelial cell migration. Angiogenesis is essential for the development of a functional corpus luteum (Reynolds et al., 2000), which is one of the most vascularized tissues in the body. Therefore, luteinization of GC should be accompanied by the up-regulation of the genes involved in angiogenic processes. By IPA analysis, HIF1a was identified to be the major upstream regulator of gene expression at LOL (**Supplementary Data Sheet S7**). HIF1a is a transcription factor, which is known to be up-regulated during luteinization in granulosa cells (Nishimura and Okuda, 2010). Upon binding within promotor regions, HIF1a induces the expression of different target genes that cumulatively induce the vascularization of tissues. Accordingly, “angiogenesis” (*p* = 1.03E-06) and “vasculogenesis” (*p* = 6.86E-07) were identified as up-regulated biological functions at LOL as revealed by IPA (**Supplementary Data Sheet S8**). HIF1a is also known to induce the glucose metabolism in different cell types (Marin-Hernandez et al., 2009). Increased glucose uptake and its metabolism have been observed in cultured murine follicles after hCG administration (Harris et al., 2007). Similarly in the present study, low oxygen levels induced the expression of *GLUT1* (Glucose transporter 1) and *GLUT 3* (Glucose transporter 3), which mediate the glucose uptake into cells. Increased expression of *GLUT1* was found in early luteal cells in response to low oxygen levels (3% O2), however, mid luteal cells failed to show such a response (Nishimura et al., 2017). In addition, substantial down-regulation of *TXNIP* expression further confirms the increased glucose metabolism at LOL. *TXNIP* is a redox sensitive signaling molecule involved in glucose metabolism (Parikh et al., 2007). Decreased expression of *TXNIP* was disclosed to be associated with increased glucose uptake in muscle and liver cells (Chutkow et al., 2008).

HBA, which encodes hemoglobin A, is majorly produced by erythrocytes. It constitutes a part of the tetrameric blood gas carrier, hemoglobin. Only a few non-hematopoietic cells are known to synthesize these proteins (Thompson et al., 2015). Interestingly, erythrocyte free HBA was identified in large antral follicles of hCG treated ovarian follicles (Thompson et al., 2015). The *HBA* gene showed a substantial up-regulation in granulosa cells cultured at LOL in the present study. Although the specific roles of hemoglobin in ovarian follicles is not yet known, identification of HBA in GC cultured at LOL further strengthens that LOL do exist in large antral follicles.

Among other important features, induction of genes associates with inflammation and leucocyte migration is widely observed in granulosa cells of preovulatory follicles (Espey, 2006; Sayasith et al., 2013). Up-regulation of such genes (*VNN1, VNN2, C1QTNF3, TNFAIP3* and *TNFAIP8* and *PTGES*) will further suggest that GC were on the verge to early luteinization and preceding ovulation at low oxygen conditions. However, the observation of no significant differences in the expression of *PTGS2*, one of the marker genes of post LH response, indicates that *PTGS2* might be regulated by LH but not by hypoxia in preovulatory granulosa cells.

A previous transcriptome analysis study describing effects of the preovulatory LH surge on antral bovine granulosa cells (*in vivo*) identified differential regulation of 2266 annotated genes upon LH surge (Christenson et al., 2013). Comparison of these genes with the present transcriptome data revealed that 1007 genes were similarly regulated in both studies but with different fold enrichment values (**Supplementary Data Sheet S9**). This important observation further strengthens the idea that prevailing low oxygen levels in preovulatory follicles could play an important role in inducing early molecular events in granulosa cells to prepare these cells for the formation of a functional corpus luteum.

Progesterone production was found to be decreased in GC under hypoxic conditions. Normally, progesterone biosynthesis is controlled by “LH receptor” (LHCGR) and finally synthesized by “hydroxy-delta-5-steroid dehydrogenase, 3 beta- and steroid delta-isomerase 1” (HSD3B1) in granulosa cells. Down regulation of these two genes was reported in GC immediately following the LH surge (Christenson et al., 2013). A similar down regulation of these two genes was observed in LOL treated GC during the present study. But, in spite of the down regulation of LHCGR and HSD3B1 expression, progesterone production was not significantly changed *in vivo* in early preovulatory ovarian follicles after LH surge (Christenson et al., 2013). In any case it needs additional studies to understand the cumulative effects of LH and hypoxia on progesterone production for further understanding of the complex *in vivo* situation.

### Cellular Proliferation Is Affected at Low Oxygen Conditions

Transformation of granulosa cells into early luteal cells is associated with decreased cellular proliferation in order to promote cellular differentiation (Stocco et al., 2007; Christenson et al., 2013; Wissing et al., 2014). Pathway analysis using IPA revealed that cellular proliferation is majorly affected at low oxygen levels. Earlier reports in humans, bovine and mice showed that expression of cell proliferation markers, *PCNA* and *CCND2*, were strongly down-regulated in follicular (preovulatory) granulosa cells that are isolated at post LH surge stages (Nimz et al., 2009; Christenson et al., 2013; Wissing et al., 2014). Similarly, LOL induced a similar phenotype in cultured GC as pathways like “Estrogen-mediated S-phase Entry,” “Cyclins and Cell Cycle Regulation” and “Cell Cycle: G2/M DNA Damage Checkpoint Regulation” were found to be significantly affected. Additionally, cell cycle inhibitors including *CDKN1A, CDKN1B*, and *CDKN1C* were also up-regulated at LOL, which is further in line with the data of Wissing et al. (2014). Down-regulation of granulosa cell proliferation was also reflected by the PPI network in which the top hub genes are *ESR1* and *KIAA0101*, both known to be involved in regulating cell proliferation in different cell types. *ESR1* encodes for the cytosolic estrogen receptor, which upon binding estrogens, translocates into the nucleus and directly binds to the DNA at estrogen responsive elements. This results in the expression of multiple target genes including genes of cell cycle progression (Oviedo et al., 2011). Therefore down-regulation of *ESR1* expression could be one major reason for the down-regulation of the estrogen mediated S-phase entry at low oxygen conditions, as revealed by IPA. Similarly *KIAA0101* is known for its strong association with *PCNA* (Emanuele et al., 2011), which plays an important role in cell proliferation.

In summary, estrogen active granulosa cells were found to remain healthy under severely low oxygen conditions and showed specific genome wide alterations associated with down-regulation of FSH signaling, cell proliferation and steroidogenesis beside up-regulation of angiogenesis, glucose metabolism and inflammatory processes.

## Conclusion

Based on the present data, we conclude that prevailing low oxygen levels in preovulatory follicles could play a key role in supporting luteinization of granulosa cells.

## Data Deposition

The microarray data were deposited in GEO by following MIAME guidelines and can be accessed with accession number GSE112070.

## Author Contributions

VB and JV designed the study. VB, AS, DK, and TV executed the experiments. VB wrote the manuscript and analyzed the data. All authors read and approved the manuscript.

## Conflict of Interest Statement

The authors declare that the research was conducted in the absence of any commercial or financial relationships that could be construed as a potential conflict of interest.

## Supplementary Material

FIGURE S1 Light microscopy pictures of cultured granulosa cells after normal and low oxygen treatments.

FIGURE S2 Signals from the 3′ and 5′ Hybridization controls in all microarray samples.

FIGURE S3 Signal box plots of all microarray samples.

DATA SHEET S1 Primers.

DATA SHEET S2 qPCR programme.

DATA SHEET S3 Total list of annotated genes and their expression values.

DATA SHEET S4 Differentially expressed genes along their expression values.

DATA SHEET S5 List of canonical pathways.

DATA SHEET S6 List of hub genes and their interactions.

DATA SHEET S7 List of upstream regulators.

DATA SHEET S8 List of biological functions.

DATA SHEET S9 List of similarly regulated genes between “post LH surge follicular granulosa cells (*in vivo*)” and “low oxygen level treated granulosa cells (*in vitro*)”.

HTML FILE S1 Interactive heat map of differentially expressed genes.

PDF FILE S1 RNA bio analyzer report.
