# Supplementary figures and images for: Low Oxygen Levels Induce Early Luteinization Associated Changes in Bovine Granulosa Cells
Source: Front Physiol. 2018 Aug 7;9:1066. doi: 10.3389/fphys.2018.01066 (PMC6090175; doi:10.3389/fphys.2018.01066)

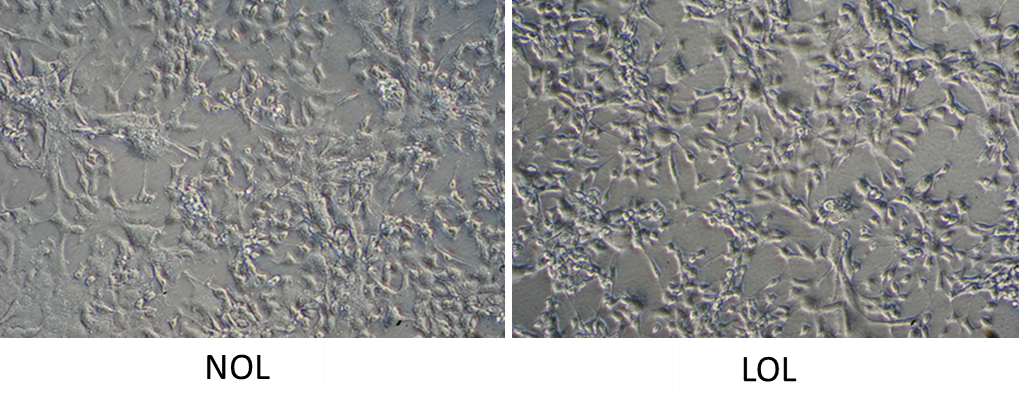

Supplement: FIGURE S1 — Light microscopy pictures of cultured granulosa cells after normal and low oxygen treatments. [file Image_1.TIF]

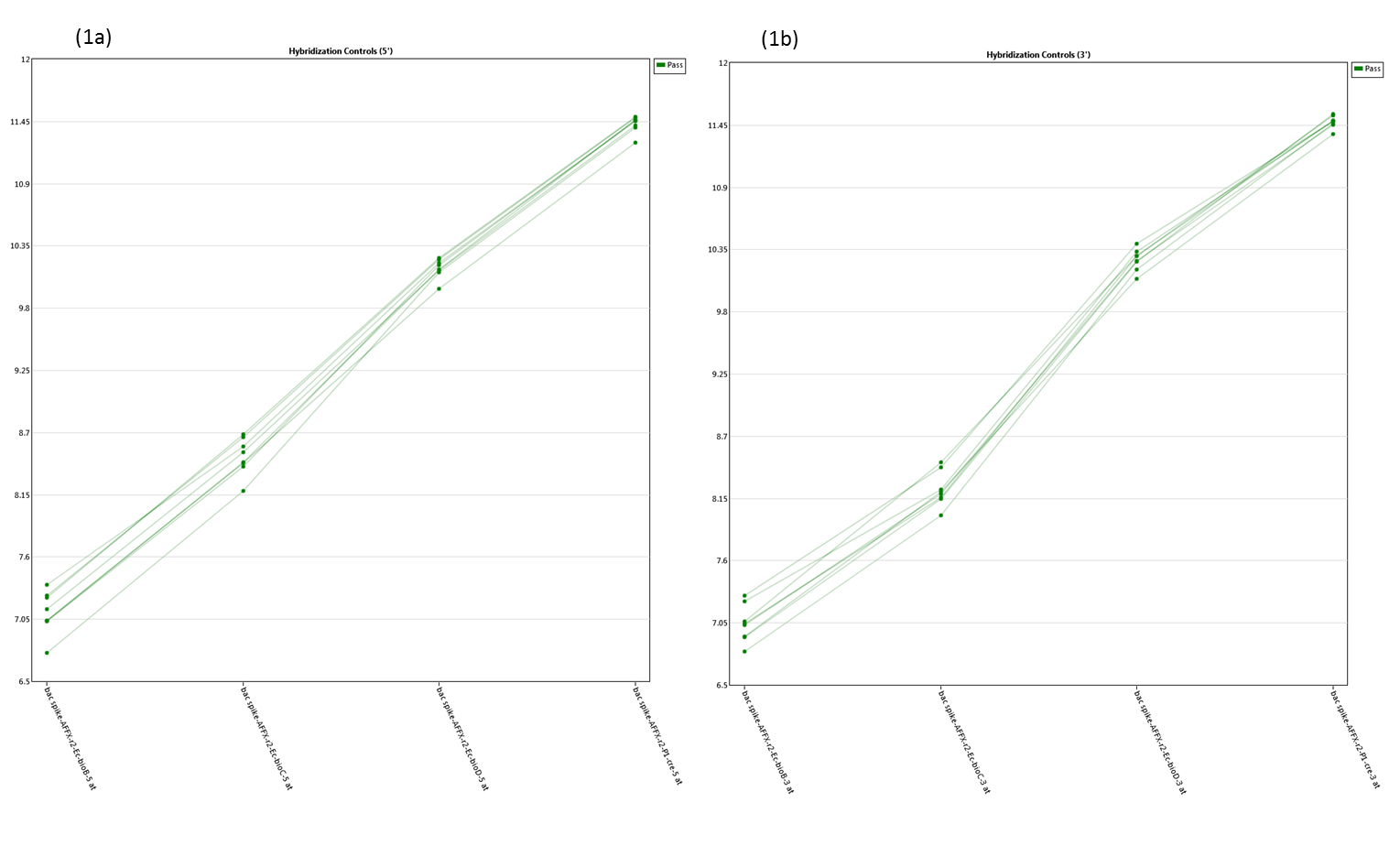

Supplement: FIGURE S2 — Signals from the 3′ and 5′ Hybridization controls in all microarray samples. [file Image_2.TIF]

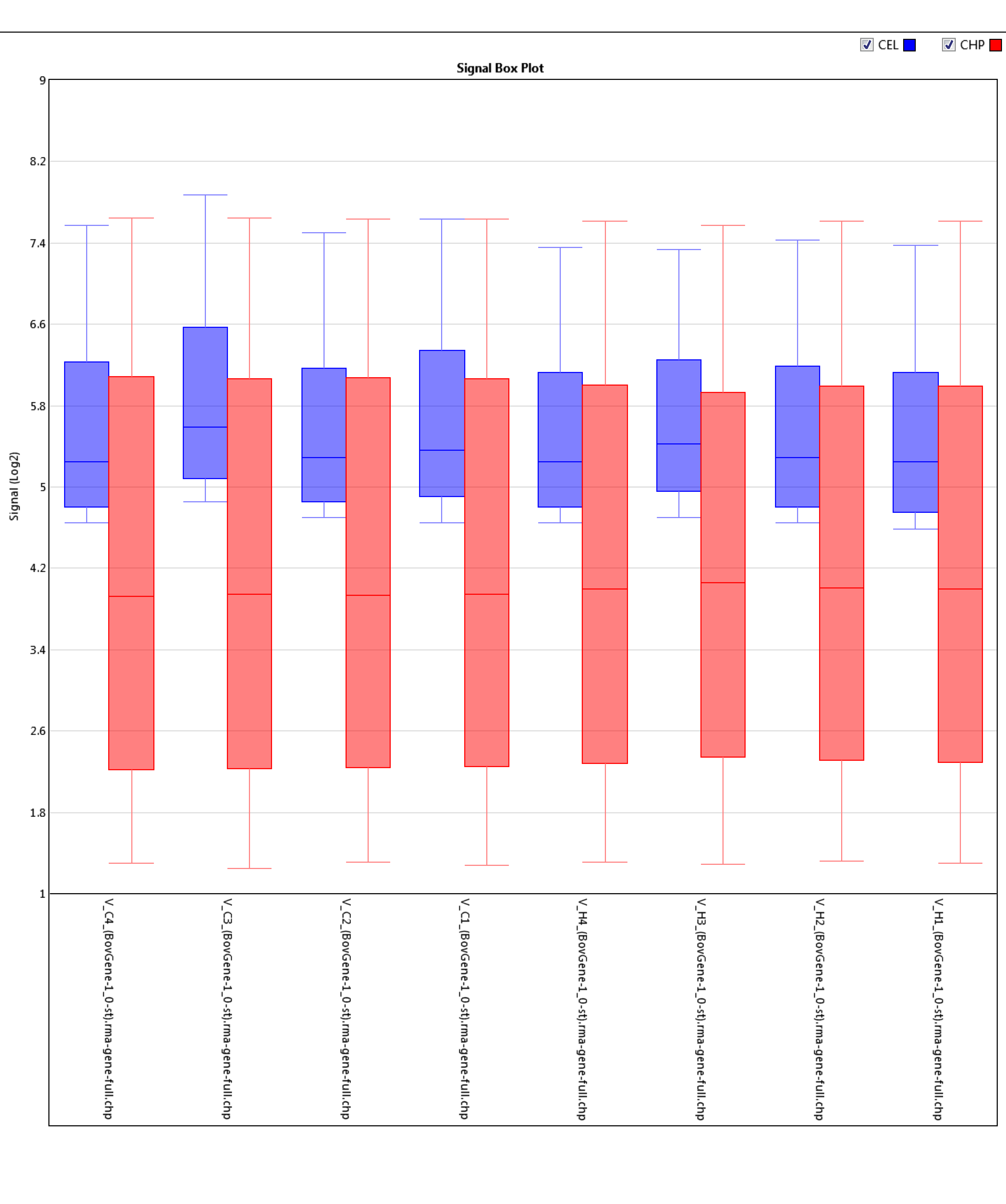

Supplement: FIGURE S3 — Signal box plots of all microarray samples. [file Image_3.TIF]
