# Supplementary material for: Low Oxygen Levels Induce Early Luteinization Associated Changes in Bovine Granulosa Cells
Source: Front Physiol. 2018 Aug 7;9:1066. doi: 10.3389/fphys.2018.01066 (PMC6090175; doi:10.3389/fphys.2018.01066)
Supplement: HTML FILE S1 — Interactive heat map of differentially expressed genes. [file Presentation_2.ZIP › supplementary html file.html]

| Parameter | Value |
| --- | --- |
| main | NULL |
| xlab | NULL |
| ylab | NULL |
| row\_text\_angle | 0 |
| column\_text\_angle | 45 |
| dendrogram | both |
| branches\_lwd | 0.6 |
| seriate | OLO |
| colors | BrBG(256) |
| distfun\_row | euclidean |
| hclustfun\_row | complete |
| distfun\_col | euclidean |
| hclustfun\_col | complete |
| k\_col | 2 |
| k\_row | 2 |
| limits | NULL |

*This heatmap visualization was created using shinyHeatmaply 2018-03-21 08:59:05*
