# Supplementary material for: Low Oxygen Levels Induce Early Luteinization Associated Changes in Bovine Granulosa Cells
Source: Front Physiol. 2018 Aug 7;9:1066. doi: 10.3389/fphys.2018.01066 (PMC6090175; doi:10.3389/fphys.2018.01066)
Supplement: PDF FILE S1 — RNA bio analyzer report. [file Presentation_1.PDF]

Assay Class: Eukaryote Total RNA Nano
Data Path: C:\...Eukaryote Total RNA Nano_DE34903669.xad

Electropherogram Summary

Ladder

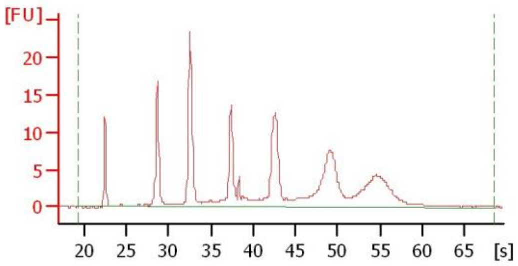

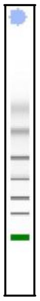

Overall Results for Ladder

RNA Area:	209.1
RNA Concentration:	150 ng/µl
Result Flagging Color:	
Result Flagging Label:	All Other Samples

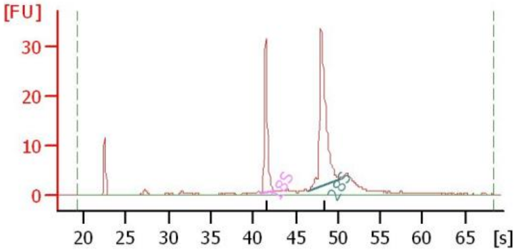

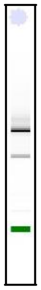

Overall Results for sample 1 : NOL1

RNA Area:	141.2
RNA Concentration:	101 ng/µl
rRNA Ratio [28s / 18s]:	1.9
RNA Integrity Number (RIN):	9.9 (B.02.08)
Result Flagging Color:	
Result Flagging Label:	RIN: 9.90

Fragment table for sample 1 : NOL1

Name	Start Time [s]	End Time [s]	Area	% of total Area
18S	40.89	42.57	28.9	20.5
28S	46.54	50.15	55.5	39.3

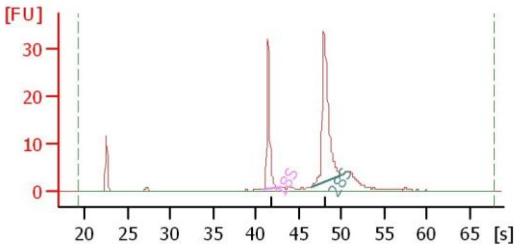

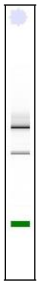

Overall Results for sample 2 : NOL2

RNA Area:	137.4
RNA Concentration:	99 ng/µl
rRNA Ratio [28s / 18s]:	1.8
RNA Integrity Number (RIN):	9.9 (B.02.08)
Result Flagging Color:	
Result Flagging Label:	RIN: 9.90

Fragment table for sample 2 : NOL2

Name	Start Time [s]	End Time [s]	Area	% of total Area
18S	40.82	42.93	30.1	21.9
28S	46.43	50.02	54.4	39.5

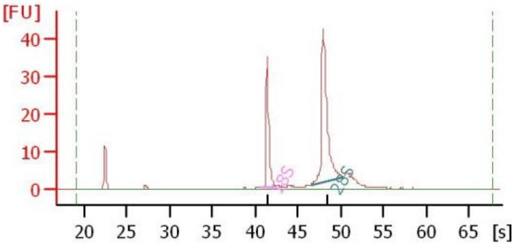

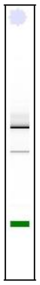

Overall Results for sample 3: NOL3

RNA Area:	152.5
RNA Concentration:	109 ng/µl
rRNA Ratio [28s / 18s]:	2.0
RNA Integrity Number (RIN):	10 (B.02.08)
Result Flagging Color:	
Result Flagging Label:	RIN:10

Fragment table for sample 3 : NOL3

Name	Start Time [s]	End Time [s]	Area	% of total Area
18S	40.78	42.45	31.5	20.7
28S	46.47	50.31	62.2	40.8

Assay Class: Eukaryote Total RNA Nano
Data Path: C:\...Eukaryote Total RNA Nano_DE34903669.xad

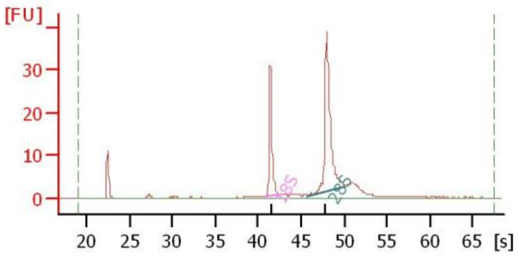

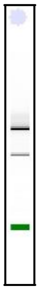

Overall Results for sample 4 : NOL4

RNA Area:	127.6
RNA Concentration:	92 ng/µl
rRNA Ratio [28s / 18s]:	2.0
RNA Integrity Number (RIN):	10 (B.02.08)
Result Flagging Color:	Result
Flagging Label:	RIN:10

Fragment table for sample 4 : NOL4

Name	Start Time [s]	End Time [s]	Area	% of total Area
18S	40.84	42.46	27.4	21.5
28S	45.78	50.14	55.1	43.2

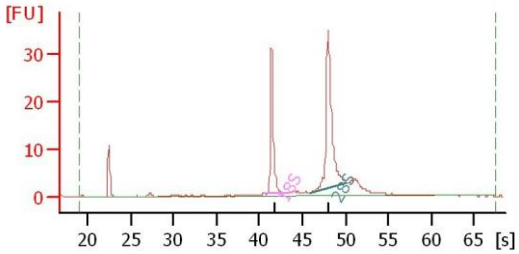

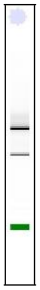

Overall Results for sample 5 : LOL1

RNA Area:	128.9
RNA Concentration:	93 ng/µl
rRNA Ratio [28s / 18s]:	1.8
RNA Integrity Number (RIN):	10 (B.02.08)
Result Flagging Color:	
Result Flagging Label:	RIN:10

Fragment table for sample 5 : LOL1

Name	Start Time [s]	End Time [s]	Area	% of total Area
18S	40.79	42.95	29.0	22.5
28S	45.83	50.09	52.4	40.6

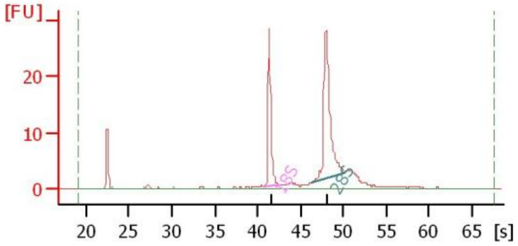

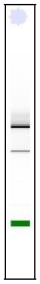

Overall Results for sample 6 : LOL2

RNA Area:	120.4
RNA Concentration:	86 ng/µl
rRNA Ratio [28s / 18s]:	1.7
RNA Integrity Number (RIN):	9.9 (B.02.08)
Result Flagging Color:	Result
Flagging Label:	RIN: 9.90

Fragment table for sample 6 : LOL2

Name	Start Time [s]	End Time [s]	Area	% of total Area
18S	40.74	42.50	27.0	22.4
28S	46.32	50.10	46.5	38.6

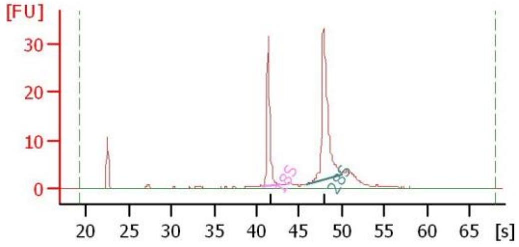

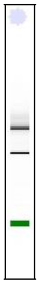

Overall Results for sample 7 : LOL3

RNA Area:	139.7
RNA Concentration:	100 ng/µl
rRNA Ratio [28s / 18s]:	1.8
RNA Integrity Number (RIN):	9.7 (B.02.08)
Result Flagging Color:	
Result Flagging Label:	RIN: 9.70

Fragment table for sample 7 : LOL3

Name	Start Time [s]	End Time [s]	Area	% of total Area
18S	40.71	42.88	28.9	20.7
28S	45.74	49.98	51.2	36.6

Assay Class: Eukaryote Total RNA Nano
Data Path: C:\...Eukaryote Total RNA Nano_DE34903669.xad

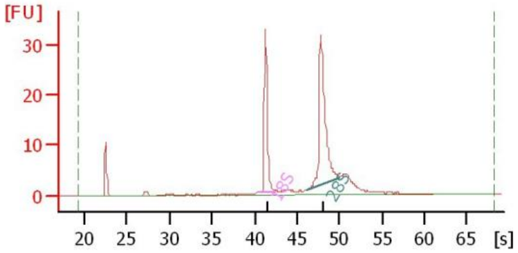

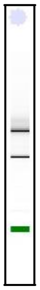

Overall Results for sample 8 : LOL4

RNA Area:	138.1
RNA Concentration:	99 ng/µl
rRNA Ratio [28s / 18s]:	1.7
RNA Integrity Number (RIN):	9.9 (B.02.08)
Result Flagging Color:	Result
Flagging Label:	RIN: 9.90

Fragment table for sample 8 : LOL4

Name	Start Time [s]	End Time [s]	Area	% of total Area
18S	40.65	42.38	30.1	21.8
28S	46.29	49.90	52.2	37.8
